# Beyond the Numbers: Assessing Clinical Risk Associated with Significant Magnesium Assay Bias

**DOI:** 10.3390/diagnostics16183029

**Published:** 2026-09-18

**Authors:** Mizanu Berihun, Kefyalew Jaleta, Shishir Adhikari, Kimberly Fankhauser, Alagarraju Muthukumar, Madhusudhanan Narasimhan

**Affiliations:** 1 William P. Jr. Clements University Hospital, University of Texas Southwestern Medical Center, Dallas, TX 75235, USA; mizanu.berihun@utsouthwestern.edu (M.B.); kefyalew.jaleta@utsouthwestern.edu (K.J.); shishir.adhikari@utsouthwestern.edu (S.A.); kimberly.fankhauser@utsouthwestern.edu (K.F.); 2 Department of Pathology, University of Texas Southwestern Medical Center, Dallas, TX 75235, USA

**Keywords:** magnesium, Mg, laboratory error, testing bias, root cause analysis (RCA), corrective and preventive action (CAPA), clinical impact

## Abstract

**Background:** Analytical variability in magnesium (Mg) assays is widely recognized, yet distinguishing its impact from intrinsic clinical risk remains under-emphasized in laboratory training. **Methods:** A routine delta check identified atypical Mg result shifts, prompting investigation and retesting on an alternate analyzer. Analytical error magnitude was quantified using absolute and percent difference and total allowable error (TE_a_), with clinical relevance assessed through integrated analytical verification, agreement analysis, and patient-level risk stratification. **Results:** A lapse in delta check containment allowed continued reporting after initial error detection. Root cause analysis (RCA) identified Mg reagent contamination and incomplete cuvette drying, causing carryover and positive bias. Among 93 samples, 59 were impacted, with deviations reaching 147.4%, and 63% (59/93) exceeded TE_a_. Despite substantial analytical bias, clinical impact was limited: 27% (16/59) remained within the reference interval and 53% (31/59) had ≤3.5 mg/dL, requiring no urgent attention. The remaining 20% (12/59) were >3.5 mg/dL, and 11/12 lacked an Mg-elevating condition (e.g., chronic kidney disease (CKD)) and required no intervention. The single CKD patient had persistently elevated Mg levels before and after correction, with no effect on interpretation, management, or monitoring. All affected results were corrected and communicated to providers. Only 1/59 (~2%) required follow-up monitoring, with no definite cause identified. **Conclusions:** This investigation demonstrates that the significance of analytical error is defined as much by clinical context as magnitude. The study identifies assay-specific and system-level vulnerabilities, particularly a gap between error detection and response. These findings underscore the need for integrated analytical, clinical, and operational safeguards and formalizing these competencies within training programs to improve laboratory quality, reliability, and practice consistency.

## 1. Introduction

Magnesium (Mg) is an essential intracellular cation involved in numerous physiological processes, including enzymatic reactions, neuromuscular function, cardiac electrophysiology, and maintenance of electrolyte balance [1]. Hypomagnesemia is commonly encountered in hospitalized patients and has been linked to cardiac arrhythmias, neuromuscular irritability, and refractory hypokalemia or hypocalcemia [2], whereas hypermagnesemia, though less frequent, may occur in renal dysfunction, in endocrine disorders such as hypothyroidism, or after excessive Mg exposure and can lead to hypotension, cardiac conduction abnormalities, and neuromuscular depression [1]. Given these clinical implications, accurate laboratory assessment of serum or plasma Mg is essential for appropriate diagnosis, monitoring, and clinical decision-making.

In routine clinical practice, unexpected and discordant Mg results are not uncommon and may arise from both pre-analytical and analytical factors. Hemolysis and Mg-containing infusions can falsely elevate results, while analytical issues such as assay interference, calibration drift, reagent instability, and instrument malfunction may introduce systematic bias [3,4,5]. When such deviations occur, it is essential not only to correct erroneous results but also to identify the underlying cause, characterize the magnitude and direction of bias, and assess the potential clinical impact.

To detect and manage such discrepancies, clinical laboratories rely on quality assurance (QA) tools such as total allowable error (TE_a_) and delta checks. TE_a_ defines acceptable limits of deviation from true values, while delta checks provide real-time comparison of current results with prior patient values to identify unexpected shifts suggestive of analytical instability or instrument malfunction. Together, these tools form a critical backbone for detecting and investigating potential laboratory errors [6,7].

While delta checks effectively identify abnormal result shifts and TE_a_ defines thresholds for quantifying analytical deviation, most prior work has focused on analytical performance metrics, with relatively limited emphasis on structured translation of analytical error into clinically actionable interpretation [6,8,9]. As a result, laboratories often operate within a gray zone, relying on professional judgment to determine the significance, urgency, and appropriate corrective actions based on potential patient impact [6].

This challenge becomes particularly evident in real-world settings, where analytical instability is first detected through delta checks, requiring a systematic investigation that extends beyond analytical verification (QC and repeat testing) to include RCA and retrospective assessment of the clinical impact on patient results that may have already been reported in the electronic health record. Yet, in practice, the skills required to apply such an integrative process are often acquired through experiential learning or informal laboratory practices rather than formalized education frameworks or fellowship-based training, contributing to heterogeneity in how such events are managed across institutions.

To address this gap, we present a structured evaluation of systematic instrument bias arising from testing issues in Mg, integrating analytical detection assessment, agreement analysis, and clinical stratification into a unified workflow. In addition to reporting an analytical error, this work provides a pragmatic educational and practice-shaping framework to support laboratory troubleshooting, clinical interpretation, and patient-centered decision-making—competencies typically honed through real-world laboratory experience.

## 2. Materials and Methods

This investigation was conducted in the university hospital’s clinical chemistry core laboratory following the identification of an unexpected increase in delta flags for plasma Mg. The study evaluated the root cause of the analytical error, the magnitude of error, the clinical impact of discrepant Mg results generated by a single affected chemistry analyzer, and the corrective and preventive measures undertaken to restore analytical accuracy and ensure sustained assay performance.

This retrospective evaluation was conducted as part of an ongoing laboratory quality assurance and quality improvement program. Analyses were performed using de-identified data generated during routine clinical operations, and no patient specimens were obtained specifically for the purpose of this assessment. The study was conducted in accordance with institutional policies governing the use of anonymized data for quality improvement activities.

### 2.1. Assay Principle, Methodology, and Detection of Analytical Error

Briefly, plasma Mg concentrations were measured on the Abbott Alinity c automated clinical chemistry analyzer using the Alinity c Magnesium Reagent Kit (Abbott Diagnostics, Abbott Park, IL, USA). Prior to analysis, lithium heparin blood samples collected as part of routine clinical care were centrifuged, and plasma was separated from cellular components by the gel barrier within the primary plasma separator tube (PST). This was then followed by automated assessments of hemolysis, icterus, and lipemia (HIL) indices and Mg measurement on the automated chemistry analyzer.

The assay is based on an enzymatic method in which Mg from the sample acts as a required cofactor for isocitrate dehydrogenase. The reagent system consists of two reagents: R1, containing isocitrate dehydrogenase (2.2 U/mL) and D-isocitrate (1.47 mg/mL), and R2, containing nicotinamide adenine dinucleotide phosphate (NADP) (8.37 mg/mL). During the reaction, Mg in the sample activates isocitrate dehydrogenase, catalyzing the conversion of D-isocitrate to 2-oxoglutarate with concomitant reduction of NADP to NADPH, as shown in the following equation. The rate of NADPH formation is directly proportional to the Mg concentration in the specimen.

(1) $$D\text{-}\mathrm{Isocitrate}+{\mathrm{NADP}}^{+}\overset{Isocitrate\;Dehydrogenase}{\underset{{\mathrm{Mg}}^{2+}}{\to }}2\text{-}\mathrm{Oxoglutarate}+{\mathrm{CO}}_{2}+\mathrm{NADPH}+H^{+}$$

For each determination, 3.2 μL of plasma was aspirated by the analyzer and mixed automatically with the assay reagents. The increase in absorbance resulting from NADPH formation was monitored kinetically at 340 nm. Mg concentrations were calculated automatically by the Alinity c analyzer using a stored calibration curve generated with the manufacturer’s multiconstituent calibrator (08P6001; Alinity c Multiconstituent Calibrator Kit, Abbott Diagnostics, Abbott Park, IL, USA). Quality control materials at normal and abnormal concentration levels (BioRad Multiqual Controls Level 1 and Level 3, BioRad, Hercules, CA, USA) were analyzed according to laboratory QC procedures to verify assay performance. Between every sample testing, the Alinity c system automatically executed probe and cuvette wash cycles, including aspiration, cleaning, rinsing, and final drying steps to avoid residual fluid and potential carryover.

The event was initially detected through routine delta check monitoring, which revealed an increase in flagged Mg results consistent with potential analytical instability. For example, an unexpectedly large increase or decrease in Mg from a previously stable value, or a series of similar abrupt increases occurring within a short period that exceeds the predefined allowable delta of 15%, warranted investigation for a potential analytical issue rather than an isolated biological change. This clustering of abnormal delta values raised suspicion for a systematic, non-random analytical shift. A technologist subsequently suspended testing on the affected instrument to prevent additional reporting of potentially erroneous results. However, a different operator inadvertently resumed the assay workflow and continued sample testing after initial issue detection.

### 2.2. Sample Selection and Data Collection

To evaluate the extent and source of the deviation, internal QC testing and reassessment of patient samples were performed, helping to distinguish potential instrument-, reagent-, or process-related causes and guiding subsequent investigation. A total of 93 patient samples analyzed during the affected time window were selected for repeat testing. All samples were reanalyzed on an alternate, unaffected chemistry analyzer under identical specimen handling conditions. Paired datasets consisting of initial (erroneous) and repeat (corrected) results were then used for subsequent analysis.

### 2.3. Analytical Comparison and Calculation

Analytical deviation between erroneous and corrected values was quantified using the following metrics:

1. Absolute difference: Erroneous – Corrected;
2. Percent difference: (Erroneous − Corrected)/Corrected × 100;
3. TE_a_ threshold: A threshold of 15% was used to define unacceptable analytical deviation based on our laboratory’s defined performance criteria, which are aligned with established biological variation-based quality specifications and are consistent with the latest available 2025 Clinical Laboratory Improvement Amendments (CLIA) allowable total error limits for Mg. Results exceeding this threshold were classified as analytically discrepant.

### 2.4. Clinical Stratification Model and Clinical Impact Assessment

**Figure 1:**
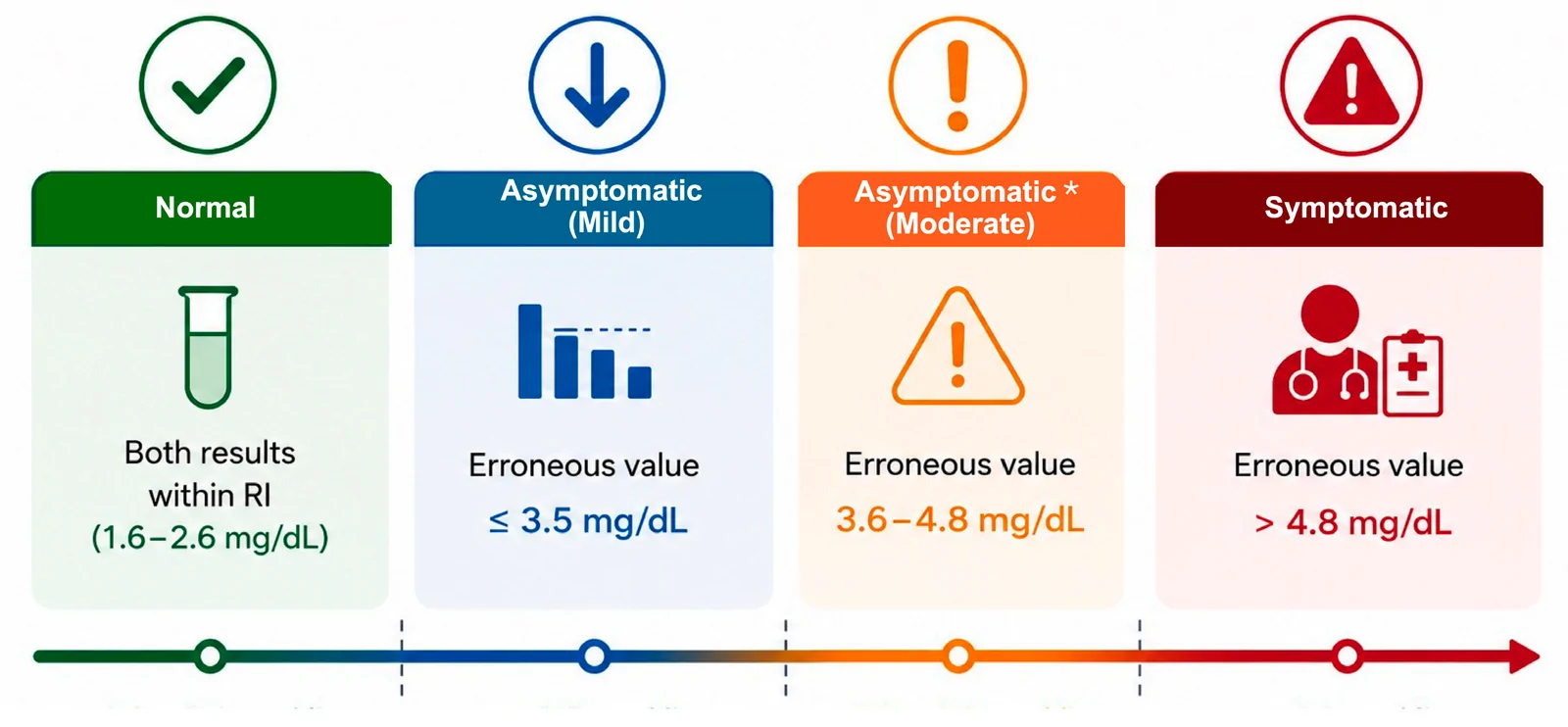
Clinical impact stratification model of Mg overestimation. * May be symptomatic in patients with chronic kidney disease (CKD). Erroneous Mg results were stratified into graded clinical risk categories based on concentration: normal (both measurements within RI), asymptomatic mild (≤3.5 mg/dL), asymptomatic moderate (3.6–4.8 mg/dL), and symptomatic (>4.8 mg/dL). This framework demonstrates increasing potential for clinical impact with higher Mg concentrations.

To translate analytical error into clinical relevance, discrepant overestimated results (*n* = 59) were categorized based on clinically relevant Mg thresholds (Figure 1). These stratification thresholds were defined based on the institutional RI for Mg and literature-supported clinical thresholds associated with symptom onset and toxicity. These categories were designed to reflect increasing clinical relevance, with mild elevations often asymptomatic (<3.5 mg/dL), and higher levels (≥4.0–4.8 mg/dL) associated with progressive clinical manifestations of moderate to high risk of hypermagnesemia [10,11]. Each discrepant result was further evaluated in the context of (i) patient-specific clinical information, (ii) presence of high-risk conditions (e.g., chronic kidney disease, hypothyroidism), and (iii) whether the discrepancy would change clinical interpretation and/or prompt intervention or additional testing. It is important to note that although hypermagnesemia is more readily recognized and monitored, the clinical impact of errors near the lower reference limit given the tight RI (0.7 to 1.7 mg/dL) may be greater. Positive analytical bias around the lower reference limit may spuriously elevate Mg and falsely normalize the results and thus could mask underlying hypomagnesemia. This will have the risk of delaying intervention and lead to related adverse clinical outcomes.

### 2.5. Root Cause Analysis and Corrective and Preventive Actions (CAPA) Evaluation

Following confirmation of systematic bias, instrument troubleshooting was initiated, and RCA was conducted to determine how erroneous results were released despite being flagged by the delta check process. A vendor consultation was sought to further evaluate potential instrument performance and maintenance-related issues. To ensure sustained quality improvement, all impacted results were reviewed and corrected where needed, and providers were notified of significant discrepancies. Ongoing monitoring included QC trend analysis, calibration profile review, reinforced delta check surveillance, and reassessment of maintenance procedures.

## 3. Results

Once discrepant Mg results were identified, we performed a structured analytical evaluation to characterize both the direction and magnitude of measurement error across the analytical range. The initial assessment focused on distinguishing systematic versus random patterns of discrepancy.

**Figure 2:**
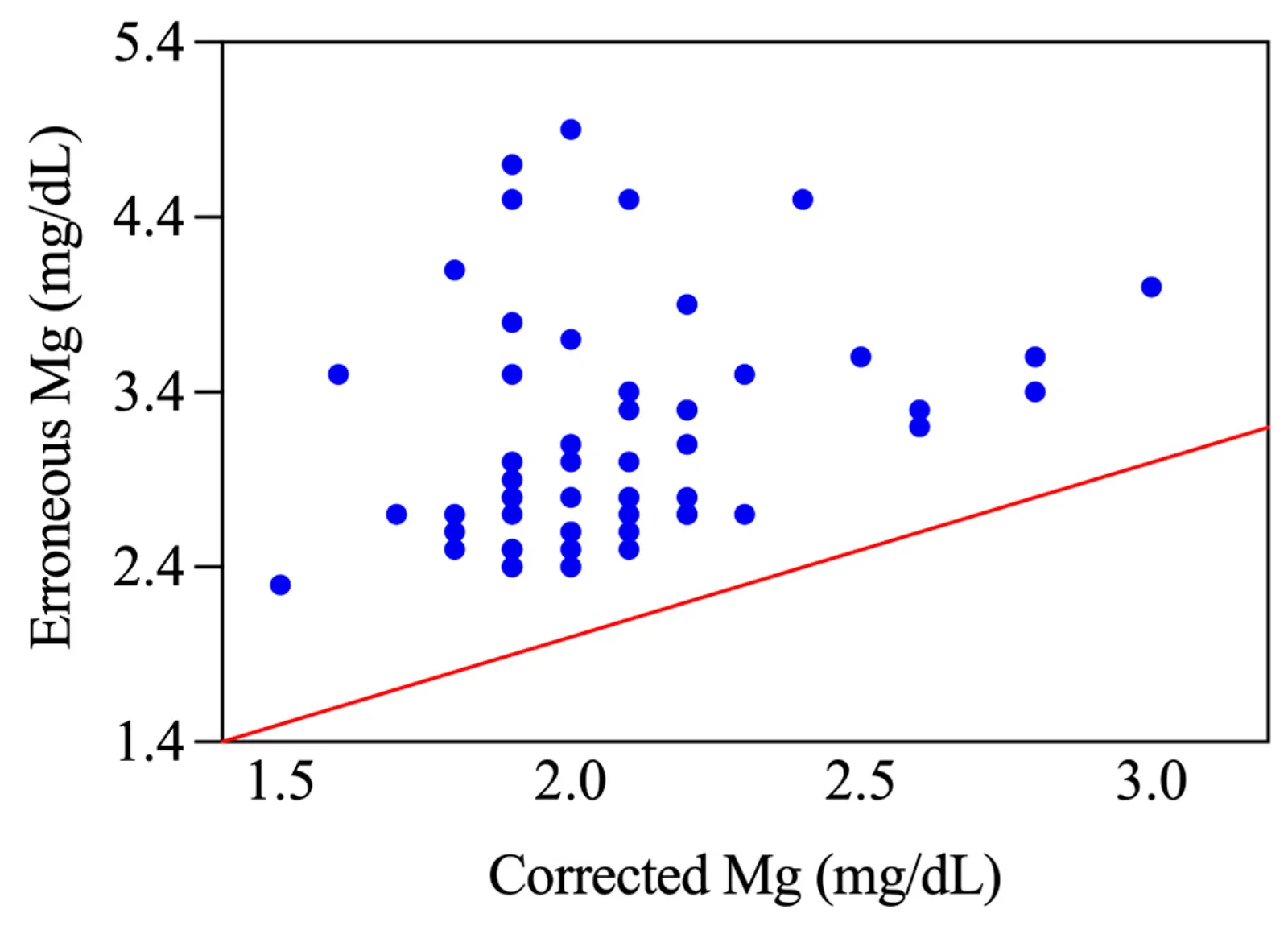
Relationship between erroneous and corrected plasma Mg values. The identity line (y = x) is shown in red. Most data points lie above this line, indicating consistent overestimation and a systematic positive bias (erroneous > corrected) across the analytical range.

Figure 2 compares corrected plasma Mg values with corresponding erroneous measurements to assess whether the affected instrument consistently overestimated Mg across the measuring range. The red diagonal line represents the line of identity (y = x), indicating perfect agreement between measurements. The clear upward shift of most points above the identity line confirms a consistent positive bias in which erroneous results systematically overestimated true values across the measurement range. This pattern was observed across the full analytical range, confirming that the overestimation was uniform rather than sporadic. The magnitude of discrepancy noted was substantial, with absolute differences of 0.4–2.9 mg/dL and percent differences ranging from 17.4% to 147.4%, highlighting significant analytical deviation. A tendency toward greater divergence at higher concentrations suggests concentration-dependent variability in bias.

**Figure 3:**
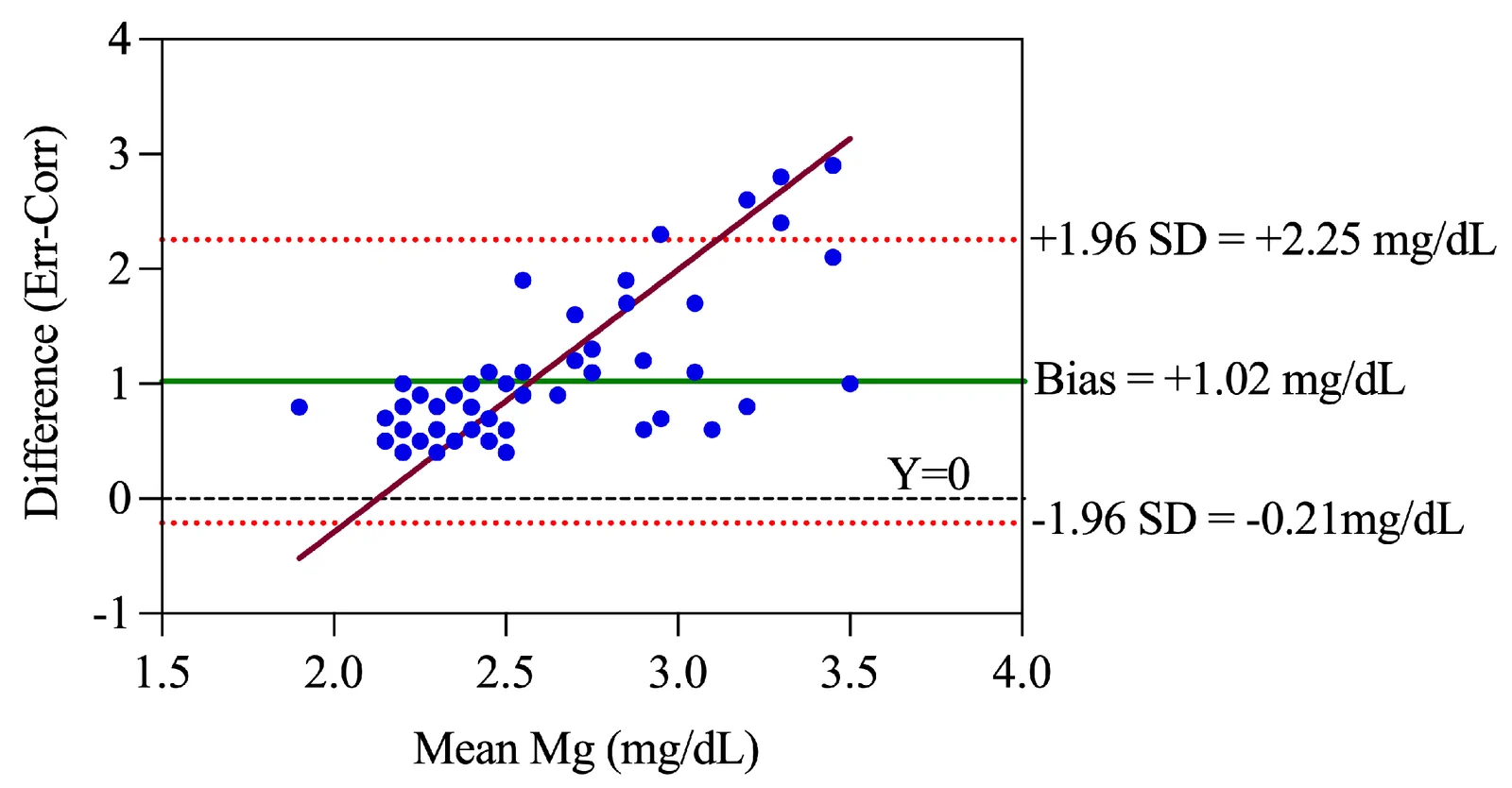
Bland–Altman assessment of Mg measurement bias. The solid green line represents the mean difference (bias) between erroneous and corrected measurements. The red dashed lines indicate the 95% limits of agreement. The black dotted line denotes perfect agreement (zero difference). The solid brown line represents the proportional bias (linear regression: Y = 1.142X − 1.928), indicating a significant increase in measurement differences with increasing Mg concentration.

Next, a Bland–Altman plot analysis was performed to evaluate agreement between erroneous and corrected Mg values by plotting the differences between paired values against their mean (Figure 3). This method provides insight into both the magnitude of systematic bias and variability of disagreement across the analytical range. Nearly all points were found to be distributed above zero, indicating consistent overestimation of erroneous values relative to corrected values and confirming a clear positive bias. The mean difference (bias) between erroneous and corrected measurements is shown by the solid green line (+1.02 mg/dL), indicating systematic positive bias by the affected assay. The limits of agreement ranged from −0.21 to +2.25 mg/dL (red dashed lines), reflecting substantial variability between measurements. The black dotted line at zero denotes perfect agreement. Regression analysis of the Bland–Altman plot revealed a significant proportional bias (brown line; slope = 1.142, 95% CI: 0.84–1.45; *p* < 0.0001), indicating that the magnitude of measurement error increased with increasing Mg concentration. The coefficient of determination (R^2^ = 0.498) suggests that approximately 50% of the variability in measurement differences was explained by concentration-dependent effects. Collectively, these findings demonstrate both systematic bias and magnitude-dependent error, with wide limits of agreement indicating poor analytical agreement. Despite the false-positive bias, no hypomagnesemia results were falsely reported as within the RI. Importantly, the absence of negative differences (values below zero) confirms a directional, systematic error rather than random measurement variation.

**Figure 4:**
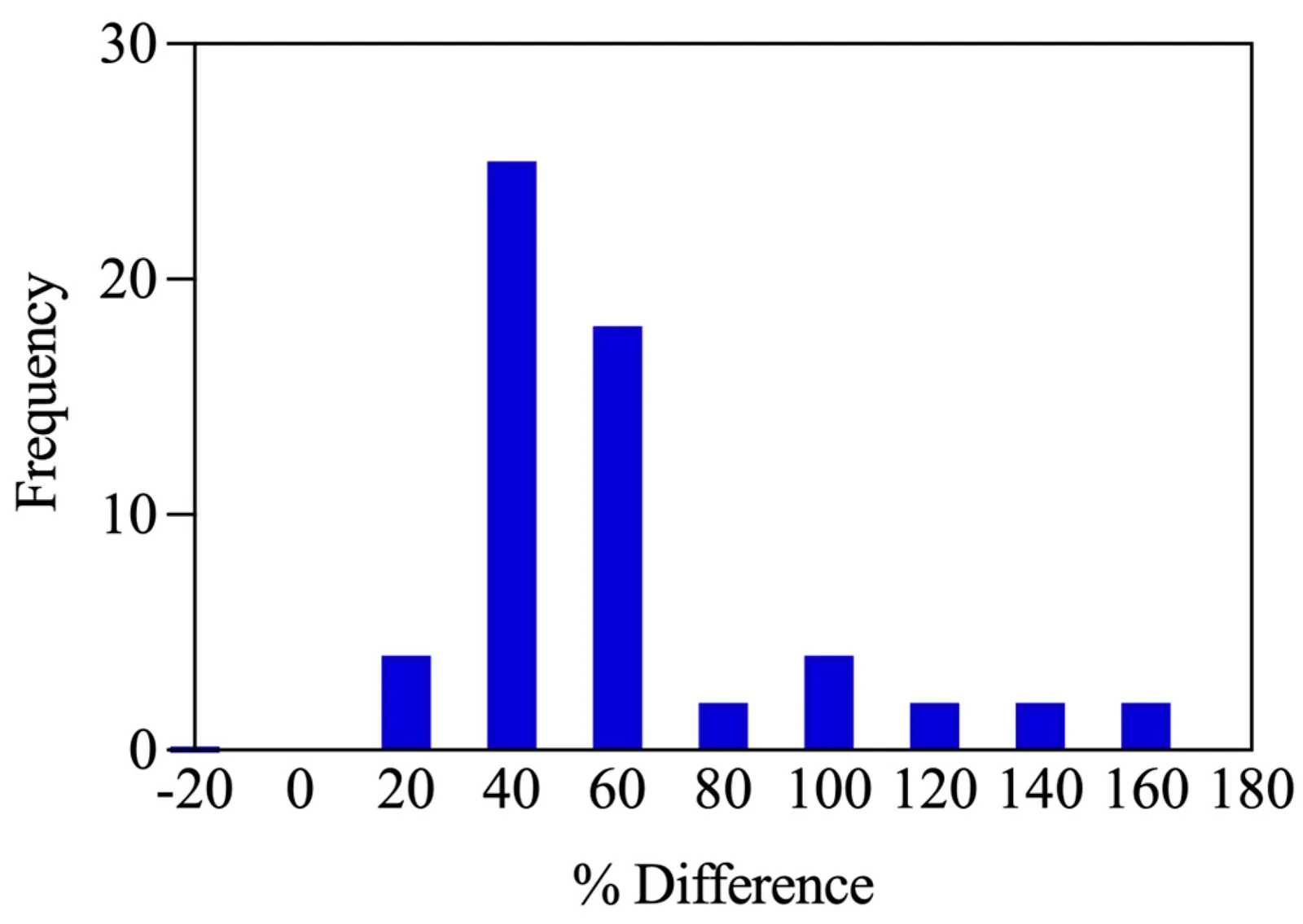
Frequency distribution of bias magnitudes in discrepant plasma Mg results. Most values cluster in the 21–60% range, with additional higher-magnitude outliers, indicating a right-skewed distribution consistent with systematic positive bias.

Next, we assessed the size of the error in percentage terms to determine the frequency distribution of discrepancies (Figure 4). The distribution demonstrated that clustering predominantly occurred in the 21–60% range, with a peak in the 21–40% bin and a secondary peak in the 41–60% range. A smaller subset extended beyond 60% and 100%, reflecting substantial analytical deviation in select cases. Notably, no negative or near-zero differences were observed, confirming uniform overestimation. Importantly, other analytes on the same platform remained stable during the affected time period. Overall, the right-skewed distribution noted here for Mg, characterized by clustering of moderate discrepancies with a tail of extreme outliers, suggests two key points: (i) the positive bias was assay-specific rather than global instrument failure, and (ii) the bias need not necessarily be trivial and may produce clinically meaningful error in select cases.

**Figure 5:**
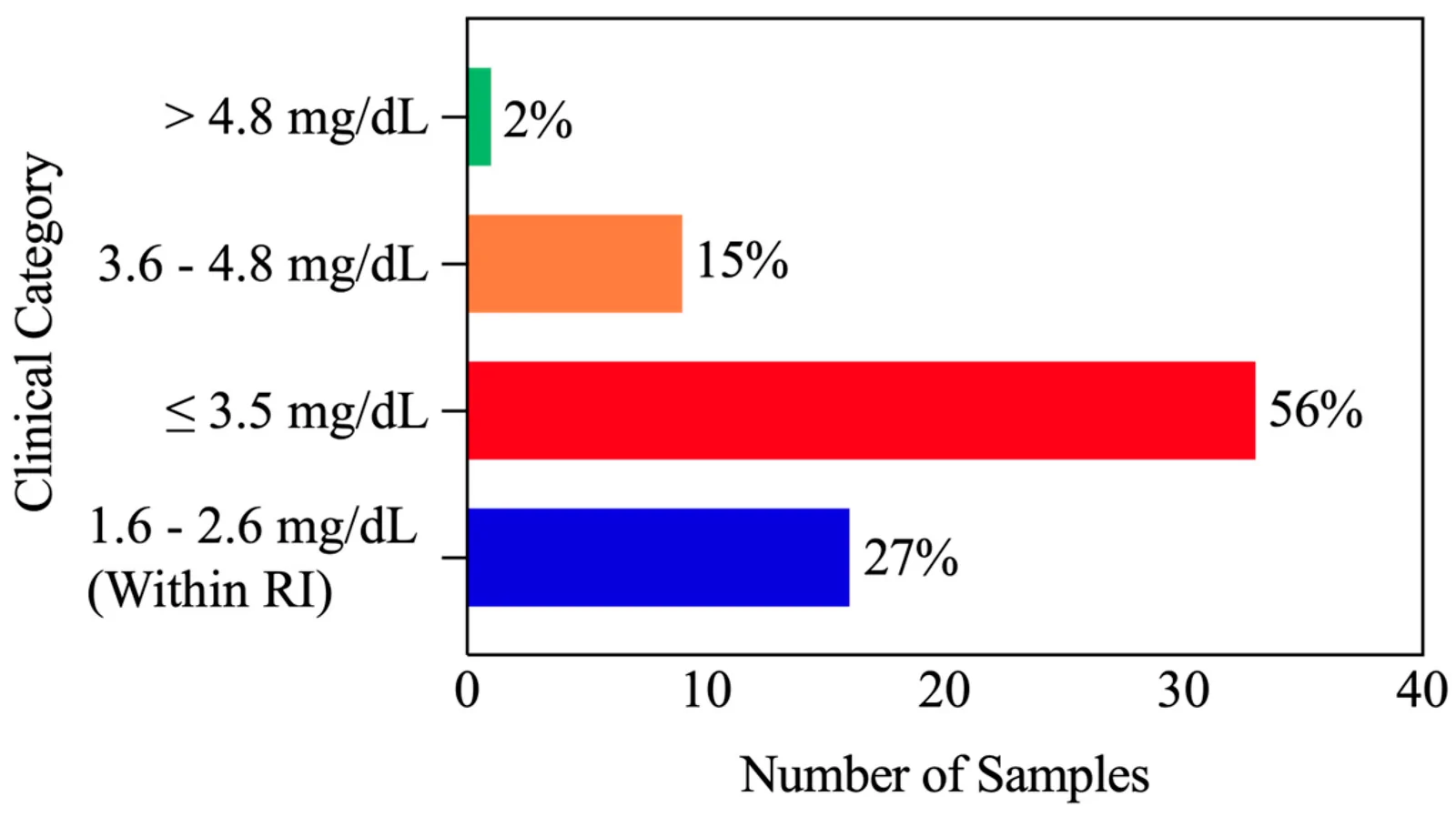
Distribution of Mg assay errors by clinical risk category. Bars represent the number of samples within each category, with percentages shown above each bar. Most discrepancies fall within ≤3.5 mg/dL, indicating limited clinical impact, while only a small proportion reaches potentially actionable ranges.

To translate the observed analytical error into a clinical context, we next performed a clinical stratification analysis of discrepant plasma Mg results (*n* = 59) using predefined clinically relevant thresholds. A smaller proportion, 27% (16/59), remained within the RI and was clinically insignificant. A further 53% (31/59) had erroneous values ≤3.5 mg/dL, which, despite exceeding analytical limits, were below clinically actionable thresholds and unlikely to influence management. Only a limited subset, 20% (12/59), exceeded the 3.5 mg/dL range, which may be considered potentially actionable primarily in CKD, a clinical condition in which Mg elevation may be more consequential (Figure 5). However, assessment of patient charts showed that 92% (11/12) were not high risk (fell within 3.6–4.8 mg/dL) and therefore required no intervention. The single patient with CKD had persistently elevated Mg levels both before and after correction, consistent with prior results; consequently, the discrepancy did not alter clinical interpretation, management, or monitoring. Only 1 of 59 results (~2%) exceeded 4.8 mg/dL; however, repeat testing yielded a result within the RI, and no identifiable contributing factor was found. Corrected results were released and actively communicated to providers in all cases without delay. Additionally, for the single case deemed clinically actionable, providers were recommended targeted monitoring and/or follow-up testing, as clinically indicated. Importantly, a post-correction review of this case indicated no alteration in care trajectory, highlighting that such large analytical discrepancies need not always translate into clinically significant impact.

**Figure 6:**
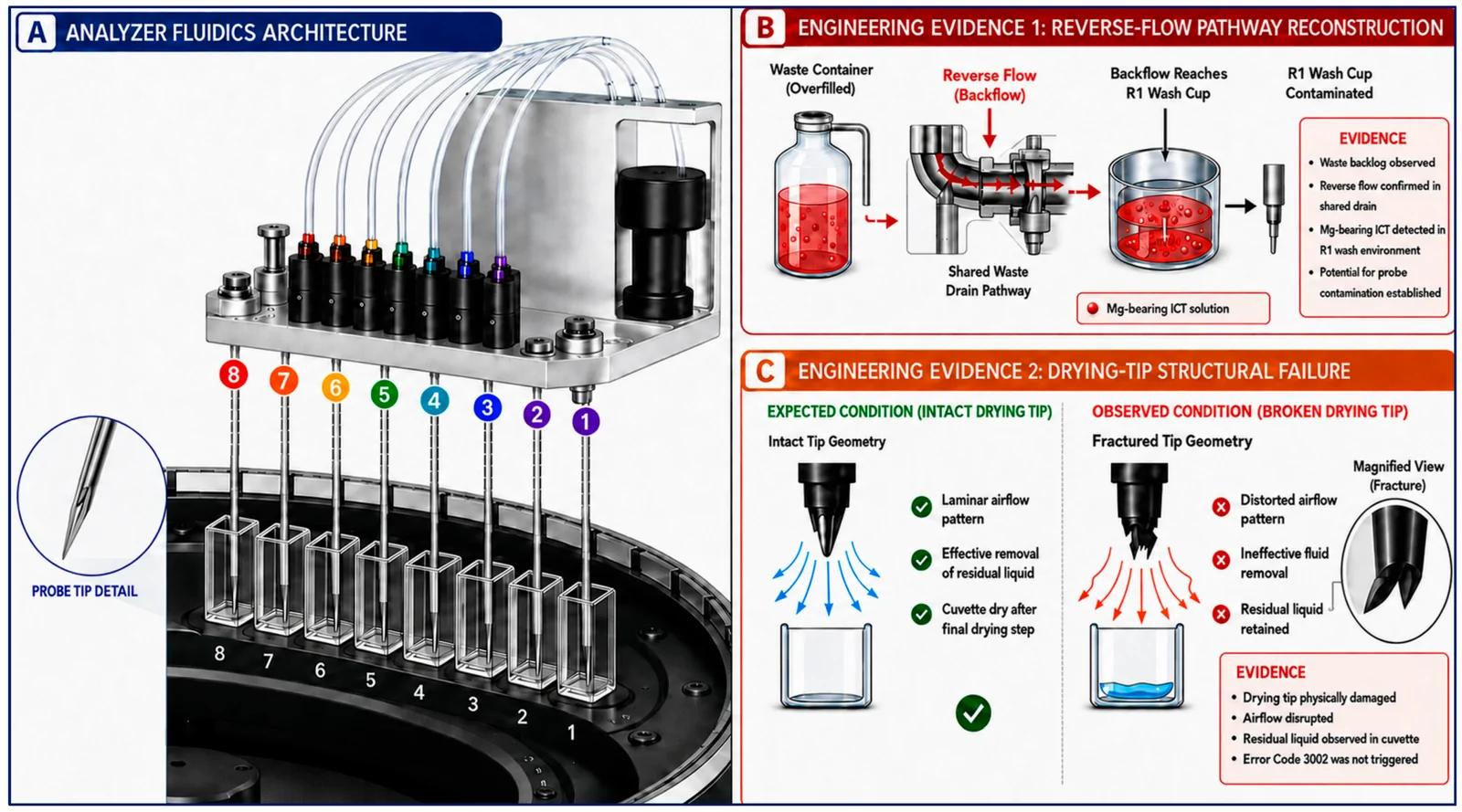
Illustration of cuvette washer architecture in the Alinity ci series chemistry analyzer and engineering evidence supporting reagent carryover. (**A**) The Alinity ci series features an integrated cuvette washer within the chemistry module, utilizing an eight-nozzle platform to aspirate, clean, rinse, and dry cuvettes before and after analytical use. The illustrated sequence highlights coordinated nozzle-driven steps, including aspiration removing residual fluid (Nozzles 1 and 7), chemical cleaning (Nozzles 2 and 3), rinsing (Nozzles 4 and 5), introducing water blank, ensuring optical integrity and confirming that cuvette is free of interfering substances (Nozzle 6), and final drying (Nozzle 8), ensuring effective removal of residual sample and reagent between cycles, minimizing carryover, and preserving analytical integrity. (**B**) Engineering evidence demonstrates reverse flow/backflow from an overfilled waste container through the shared drain pathway, creating a backlog reaching and contaminating the R1 wash cup with Mg-bearing fluid. (**C**) Structural inspection identified a fractured drying-tip geometry, resulting in disrupted airflow (schematically shown by red arrows), incomplete residual fluid removal, and retained liquid within the cuvette. Together, the findings support a combined reverse-flow and drying-tip failure mechanism leading to reagent carryover and the observed analytical bias.

Root cause analysis identified that the Mg bias resulted from a multifactorial mechanism involving mechanical, fluidic, reagent, and operational factors. In particular, we identified two parallel sources of analytical issues contributing to the observed Mg bias: (i) R1 probe carryover due to waste-fluid contamination, and (ii) incomplete cuvette drying caused by a missing drying-tip component. The Abbott Alinity c system includes several built-in safeguards and error codes programmed to alert the users when such safeguards are compromised; however, no specific error code exists to detect R1 probe contamination arising from this waste fluid backflow issue. The cuvette washer operates through an eight-step sequence of aspiration, cleaning, rinsing, and final drying between tests. The final drying step is particularly critical, as it removes residual liquid from the cuvette and prevents carryover (Figure 6).

In this case, a backlog in the liquid waste container caused waste to flow back into the Reagent R1 probe wash station via a shared waste drainage pathway. Notably, despite the Abbott Alinity c system being equipped with multiple sensors and error detection mechanisms to prevent overflows before impacting patient results, no alert/error code was triggered for this waste backup event. The waste stream contained Mg-bearing ICT solution, and backflow into the R1 wash cup resulted in enrichment of the wash fluid with trace Mg. Thus, intermittent contact between the R1 probe and this Mg-contaminated waste likely led to probe-tip contamination, followed by reintroduction of trace Mg into the reaction pathway, producing probe-mediated carryover.

Concurrently, we identified a broken cuvette drying tip that could compromise the final cuvette-drying step, allowing residual wash effluent and remnants of prior reactions to persist within the cuvette. Although the system includes a specific built-in error code (3062) to detect residual liquid following the drying step, no alert was triggered in this instance. The interplay of these two defects amplified the analytical impact: probe-mediated introduction of Mg increased the contaminant load, while ineffective drying prevented its removal, allowing Mg-enriched residual fluid to persist and carry forward into subsequent measurements. This effect was most pronounced for the Mg assay, which is particularly sensitive to contamination and carryover. The assay protocol therefore incorporates additional Smart Wash cycles when certain analytes are run before or after it, increasing the burden on the wash system. Additionally, the assay is sensitive due to utilization of low sample volume and its narrow RI. Given these assay characteristics, any issues in washing or drying as observed here can disproportionately affect Mg results and lead to significant positive bias. While other assays requiring low sample volumes (e.g., calcium and bilirubin) could theoretically be impacted by incomplete cuvette washing and R1 probe carryover, our patient result assessment showed no measurable impact on these analytes. In parallel, operational factors, including communication breakdowns and competing workflow priorities, contributed to continued result reporting despite early abnormal delta flags. Although these operational factors do not directly contribute to the analytical error, they hindered its timely detection and response. Thus, in summary, the analytical factors explained the origin of bias, whereas operational factors accounted for their persistence.

## 4. Discussion

### 4.1. Error Detection and Initial Response

This delta check-triggered investigation demonstrates a systematic approach to identifying and managing plasma Mg overestimation in a clinical laboratory. The earliest signal of error was a rise in delta flags for Mg, prompting immediate investigation. Testing was suspended and QC evaluation initiated, confirming an isolated, instrument- and assay-specific issue rather than a systemic issue affecting several assays or instruments. Early containment is particularly critical in Mg testing, where inaccurate overestimation or underestimation may directly influence clinical interpretation and patient management [12]. This scenario further reinforces delta checks as a sensitive complementary tool to routine QC for early detection of analytical instability in high-throughput settings [13].

### 4.2. Analytical Confirmation and Bias Characterization

Analytical verification through QC failure and parallel testing on an alternate analyzer confirmed a systematic positive bias specific to the Mg assay on a single instrument. Importantly, 63% of results exceeded the 15% TE_a_ threshold, with deviations reaching as high as 147.4%, and the bias remained consistently directional across the analytical range (erroneous > corrected). The significant proportional bias observed (R^2^ = 0.498, *p* < 0.0001) demonstrates that error magnitude increased with higher Mg concentrations, supporting that the nature of the error is not random [14]. Notably, these findings highlight the value of cross-instrument verification as a practical approach for confirming suspected analytical bias. In routine laboratory practice, such comparative evaluation serves as a critical safeguard, enabling timely identification of potential instrument-specific errors and supporting informed decision-making in quality management.

### 4.3. Root Cause Resolution

Initial corrective actions, including replacement of the drying tip and wash tubing, failed to restore QC performance. Only replacement of the specific Mg reagent cartridge followed by recalibration restored QC, indicating secondary reagent compromise. The absence of abnormalities in Ca, bilirubin, and alkaline phosphatase (ALP) results supports an assay-specific issue rather than a broad systemic failure. Together, these observations indicate a multifactorial mechanism involving mechanical, fluidic, and reagent-level contributions. A key takeaway from this investigation is that unresolved or frequent QC issues following mechanical troubleshooting should prompt a concurrent reagent integrity evaluation alongside careful interpretation of the recalibration curve. Specifically, calibration factors and curve characteristics should be assessed in the context of the laboratory’s historical performance to identify subtle, unexpected inconsistencies. In this case, QC values were brought within acceptable ±2 SD limits after recalibration; however, a positive bias in patient results persisted, as recalibration had been performed using a compromised reagent pack. This discrepancy led to closer scrutiny of the calibration curve, which revealed marked deviation from the expected curve characteristics. Replacing the contaminated reagent pack and fresh calibration ultimately restored calibration reliability, assay performance, and patient result accuracy.

This event additionally revealed a gap in our operational response to analytical error detection. Despite identification of the issue via delta checks and suspension of the affected assay in that specific instrument, a communication breakdown allowed results to continue being released inadvertently. Our RCA identified that variability in staff expertise, concurrent workload demands, turnaround time expectations, and communication barriers between technologists and across shifts likely contributed to issues typical in high-volume laboratory settings.

To address the identified operational gaps, targeted quality improvement measures are being considered, including analyzer lockout mechanisms, enhanced prioritization of alerts within the laboratory information system (LIS), improved documentation of overrides, additional staff training, and more structured communication practices. In parallel, the instrument vendor will be engaged to assess current limitations in error code generation and explore potential system-level solutions, such as improved alert functionality or configuration adjustments. Together, these efforts aim to promote consistency, reduce reliance on individual judgment, and prevent the continuation of compromised testing, while carefully balancing escalation needs with alert fatigue.

### 4.4. Translating Analytical Error into Clinical Context

A central finding of this study lies in differentiating analytical deviation from true clinical relevance. Despite identifying a substantial bias exceeding established TE_a_ limits, the actual impact on patient care was limited, as most discrepancies remained below actionable thresholds. Even when values appeared potentially concerning, clinical decisions were unaffected due to the patient’s pre-existing conditions. These findings reinforce that analytical error magnitude alone is not a reliable indicator of clinical risk; rather, its significance is determined by its relationship to assay-specific decision thresholds, patient risk factors, and the broader clinical context.

The existing literature has documented multiple sources of Mg variability, including pre-analytical factors such as hemolysis and analytical factors related to instrumentation and reagents [3,4,5,15]. Instrument-specific biases, often stemming from mechanical and optical limitations, are well documented [16,17,18]. However, these studies have largely been confined to analytical performance evaluation without addressing clinical translation [19,20]. Consequently, this gap is critical, as both overemphasizing flawed results and overlooking detection of meaningful errors can both pose risks to patient safety. The present study addresses this need by coupling quantitative error assessment with structured clinical stratification, reinforcing that the relevance of laboratory error is inherently contextual rather than purely numerical.

### 4.5. Practical Framework for Managing Overestimation of Mg in Laboratory Practice

**Figure 7:**
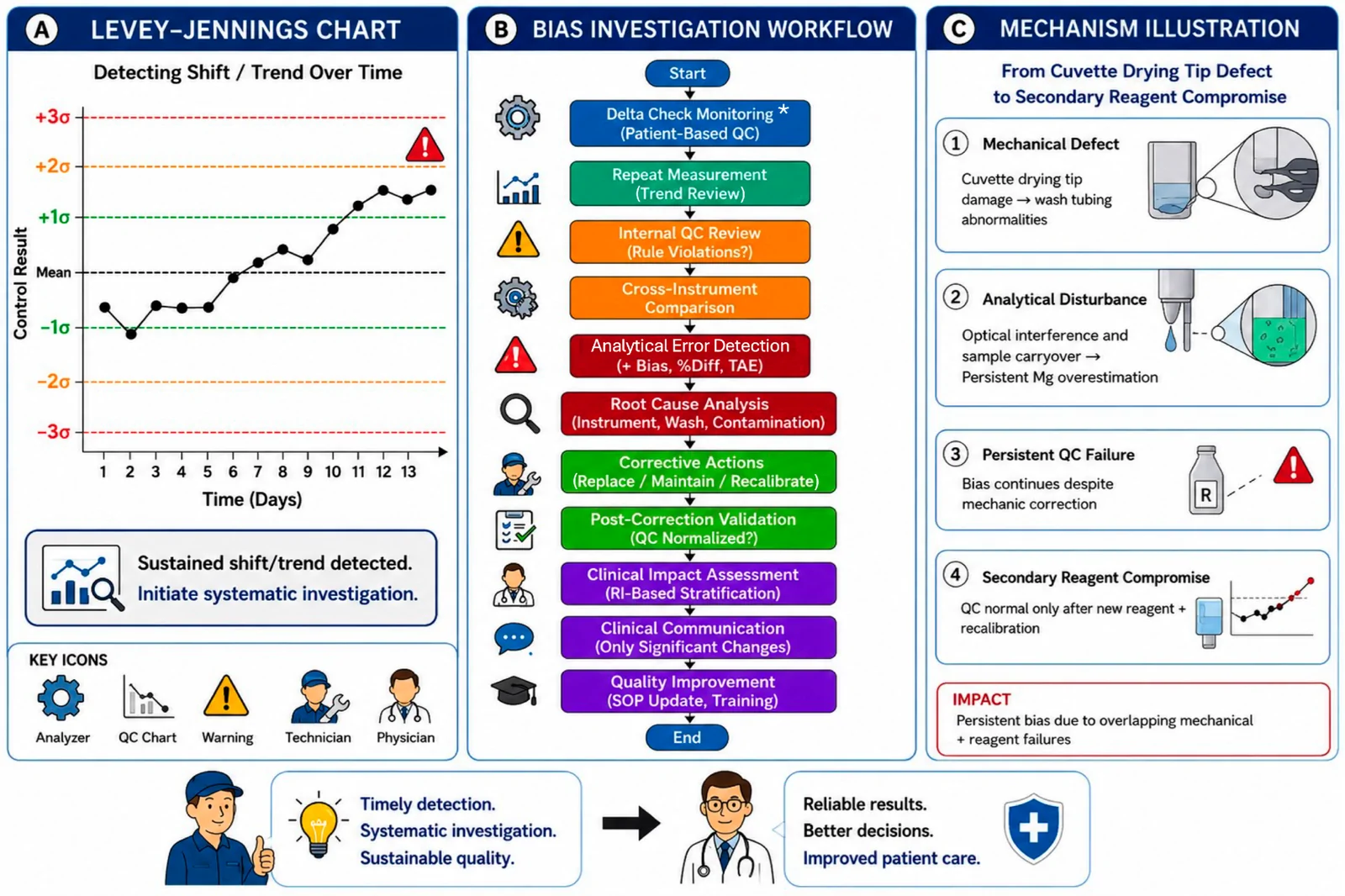
Integrated framework for detection, investigation, and mechanistic characterization of analytical bias linked to Mg overestimation. (**A**) Conceptual Levey–Jennings plot showing a sustained upward trend used for illustrative purposes (non-data derived). (**B**) Stepwise workflow for bias investigation and clinical impact assessment. (**C**) Mechanistic pathway linking cuvette drying defects to reagent-mediated bias. * Where applicable.

The present work provides several key transferable learning points that can be operationalized into a practical, stepwise workflow for managing suspected Mg-related analytical bias (Figure 7). Importantly, this workflow can be tailored contextually and is broadly applicable to other assays that are prone to errors.

1. Detection: Monitor delta flags and QC trends to identify early analytical instability.
2. Immediate Containment: Suspend testing promptly and verify QC to prevent further erroneous reporting.
3. Confirmation: Perform cross-platform comparison using patient samples to quantify bias direction and magnitude.
4. RCA: Assess reagent integrity, calibration status, and instrument fluidics, including assessment for mechanical issues.
5. Clinical Triage: Stratify affected results using clinical thresholds and prioritize review based on patient context and risk.
6. Communication and Documentation: Issue corrected reports as needed, notify providers and offer retesting when appropriate, and ensure thorough documentation in accordance with laboratory quality standards.

### 4.6. Limitations

This single-center study may not generalize to other laboratories, platforms, reagent lots, or workflows, where differences in instrument design, calibration, maintenance, workload, staffing, and testing processes may affect both analytical failures and their detection. Multicenter studies should validate the failure mechanism and evaluate standardized surveillance approaches besides delta checks—moving averages and autoverification limits—using probability of error detection (Ped), results-to-detection metrics, and false-positive rates. Thresholds should be locally validated to balance detection sensitivity against alert burden and manual review. AI-based methods may further enhance early detection by integrating patient, instrument, and QC data.

Clinical impact was assessed retrospectively and may underestimate unrecognized provider responses or downstream consequences. Furthermore, the observations are specific to plasma Mg testing and may not extend to tests with different analytical or clinical characteristics. Notably, the investigation also did not incorporate moving average–based trend analysis such as exponential moving average (EMA). Future approaches may benefit from incorporating EMA surveillance, which could enhance early detection of subtle systematic shifts that are not captured by conventional QC or delta check mechanisms.

## 5. Conclusions

A delta check-triggered investigation into discrepant plasma Mg results uncovered a systematic positive bias and highlighted a broader challenge in diagnostic medicine: quality systems are often designed to detect error but not necessarily to ensure its timely resolution. Breakdowns in communication, escalation, and workflow response allowed the issue to persist beyond initial recognition. Structured analytical verification, root cause analysis, and clinical stratification confirmed that most discrepant results had limited clinical impact. These findings reveal a systems-level gap between surveillance and response, underscoring that diagnostic safety relies not only on analytical robustness but also on the ability to rapidly translate detection into action. Beyond immediate lessons of this event, we propose a practical framework for integrated error management and advocate for embedding systems-based approaches to anomaly detection, investigation, and mitigation into formal laboratory medicine education and fellowship training. Strengthening these competencies will be essential for improving responsiveness and resilience in increasingly complex testing environments.

## Data Availability

De-identified data supporting the conclusions of this article will be made available by the authors on request.

## References

[1] Kothari M., Wanjari A., Shaikh S.M., Tantia P., Waghmare B.V., Parepalli A., Hamdulay K.F., Nelakuditi M. (2024). A Comprehensive review on understanding magnesium disorders: Pathophysiology, clinical manifestations, and management strategies. Cureus.

[2] Salinas M., López-Garrigós M., Flores E., Leiva-Salinas C. (2023). Improving diagnosis and treatment of hypomagnesemia. Clin. Chem. Lab Med..

[3] Lameris A.L., Monnens L.A., Bindels R.J., Hoenderop J.G. (2012). Drug-induced alterations in Mg homoeostasis. Clin. Sci..

[4] Ma C., Li X., Luo W., Hou L., Sun D., Liu L., Liu X., Zhang Y., Xu J., Qiu L. (2026). Real-world study: Assessing the impact of hemolysis on 48 biochemical and immunological analytes through big data analysis and its feasibility validation. PLoS ONE.

[5] Ab Rahim S.N., Nordin N., Wan Omar W.F.A., Zulkarnain S., Kumar S., Sinha S., Haque M. (2023). The laboratory and clinical perspectives of magnesium imbalance. Cureus.

[6] Campbell M.R., Galior K. (2026). An overview of allowable total error in the clinical laboratory. J. Appl. Lab Med..

[7] Castro-Castro M.J., Sánchez-Navarro L. (2021). Estimation of change limits (deltacheck) in clinical laboratory. Adv. Lab Med..

[8] Karger A.B. (2017). To delta check or not to delta check? That is the question. J. Appl. Lab Med..

[9] Schifman R.B., Talbert M., Souers R.J. (2017). Delta Check Practices and Outcomes: A Q-probes study involving 49 health care facilities and 6541 delta check alerts. Arch. Pathol. Lab Med..

[10] Yu A.S.L., Gupta A. Hypermagnesemia: Clinical Manifestations and Management. UpToDate.

[11] Bringhurst F.R.D.M., Krane S.M., Kronenberg H.M., Longo D.L.F.A., Kasper D., Hauser L., Jameson S.L., Loscalzo J.L. (2012). Bone and mineral metabolism in health and disease. Harrison’s Principles of Internal Medicine.

[12] Costello R.B., Nielsen F. (2017). Interpreting magnesium status to enhance clinical care: Key indicators. Curr. Opin. Clin. Nutr. Metab. Care.

[13] Yang J., Wen S., McCudden C.R., Tacker D.H. (2024). Selection of Single-Analyte Delta Check Rules with Logistic Regression for Detection of Intravenous Fluid Contamination in a Clinical Chemistry Laboratory. J. Appl. Lab Med..

[14] Mulya Harahap R.I., Kurniati I., Suraya N., Rostini T., Ropii B., Bethasari M. (2025). Comparative Analysis of serum magnesium ion levels using three measurement methods: Spectrophotometry, atomic absorption spectrophotometry, and inductively coupled plasma with optical emission spectrophotometry. Int. J. Anal. Chem..

[15] Ge M., Zhao H., Yan Y., Zhang T., Zeng J., Wang Y., Meng Q., Zhang C. (2016). Evaluation of the bias of serum magnesium measurements and the commutability of processed materials. Clin. Lab.

[16] Westgard J.O. (2010). Managing quality vs. measuring uncertainty in the medical laboratory. Clin. Chem. Lab Med..

[17] Song X.D., Li S.X., Qin Z.M., Chen D.L., Guo L.L., Liu C.R., Yang X., Peng K.N., Dai E.H. (2024). Ensure the accuracy and consistency of biochemical analyzer test results: Chemometrics for instrument and inter-instrument item comparison in Chinese hospital laboratory. Heliyon.

[18] Boneno J., Fokakis M., Armbruster D. (2005). Reagent carryover studies: Preventing analytical error with open clinical chemistry systems. Lab Med..

[19] Smith A.F., Shinkins B., Hall P.S., Hulme C.T., Messenger M.P. (2019). Toward a framework for outcome-based analytical performance specifications: A methodology review of indirect methods for evaluating the impact of measurement uncertainty on clinical outcomes. Clin. Chem..

[20] Gounden V., Banerjee M., Amundsen E.K., Serdar M.A., Suárez Sánchez C.I., Strain C., Kinniburgh D., Zhao Z. (2023). Linking laboratory testing to clinical outcomes: Bridging the gap through outcome-based studies in laboratory medicine. Clin. Chem..

